# Hot to touch: the story of the 2021 Nobel Prize in Physiology or Medicine

**DOI:** 10.1242/dmm.049352

**Published:** 2021-10-27

**Authors:** Darren W. Logan

**Affiliations:** Waltham Petcare Science Institute, Freeby Lane, Waltham on the Wolds, Leicestershire LE14 4RT, UK

## Abstract

The 2021 Nobel Prize in Physiology or Medicine was awarded to Ardem Patapoutian and David Julius for their research on receptor channels responsible for the perception of touch and temperature. Somatosensation, an overarching sense that enables us to safely interface with the physical forces around and within us, is the fourth sensory modality to be recognized by the Nobel Committee. The story of the discovery of TRP and PIEZO channels, and subsequent investigations into their myriad roles in the perception of noxious and mild temperature, touch, pain, pressure and body position, is an archetype for how translational research into human and animal health is built on a foundation of excellence in basic science.

## Abstract (precis)

**Summary:** This year’s Nobel Prize in Physiology or Medicine celebrates how even the most fundamental of science can lead to broad applications for improving health and wellbeing.

Appropriately sensing the world around us is fundamental to our wellbeing. From avoiding spoilt food, and seeing and hearing an approaching car, to smelling leaking gas or feeling the searing heat of a burning stove, our senses work together to keep us alive. It is perhaps unsurprising, therefore, that the research that revealed the molecular and cellular mechanisms underpinning our senses has repeatedly attracted the attention of the Nobel Committee in Physiology or Medicine. Hearing (1961), sight (1967) and smell (2004) have all been recognized previously. This year, the Nobel Prize in Physiology or Medicine was awarded to two scientists, Drs David Julius and Ardem Patapoutian, for their discovery of the receptors that enable the detection of temperature and touch.

The origins of their work can be traced to another pair of Nobel winners. In 1944, Drs Joseph Erlanger and Herbert Gasser were awarded the same prize for their work on the formation of action potentials in different types of peripheral nerve fibres. The peripheral neurons they described are responsible for transmitting the sensation of pain, but how does a neuron translate a noxious physical force, such as thermodynamic energy or pressure, into an action potential?

In the 1990s, David Julius sought to answer this question by exploiting an unusual characteristic of some naturally occurring compounds – namely, that they are perceived as having temperature. Capsaicin, responsible for the hot sensation associated with eating chillies (Fig. 1), is perhaps the most famous of these chemicals. Taking a high-risk brute-force approach, Julius and colleagues at the University of California, San Francisco (San Francisco, CA, USA) heterologously expressed pools of thousands of complementary DNA (cDNA) clones from dorsal root ganglia in embryonic kidney cells and screened for capsaicin-evoked changes in intracellular calcium (Caterina et al., 1997). After many serial dilutions of clone pools followed by calcium imaging, they ultimately identified a mammalian homologue of the transient receptor potential (Trp) ion channel first identified in *Drosophila melanogaster* photoreceptors. They demonstrated that the channel, later named TRPV1, was uniquely expressed in thermosensory neurons and responsive to both capsaicin and noxious heat.

**Fig. 1.**
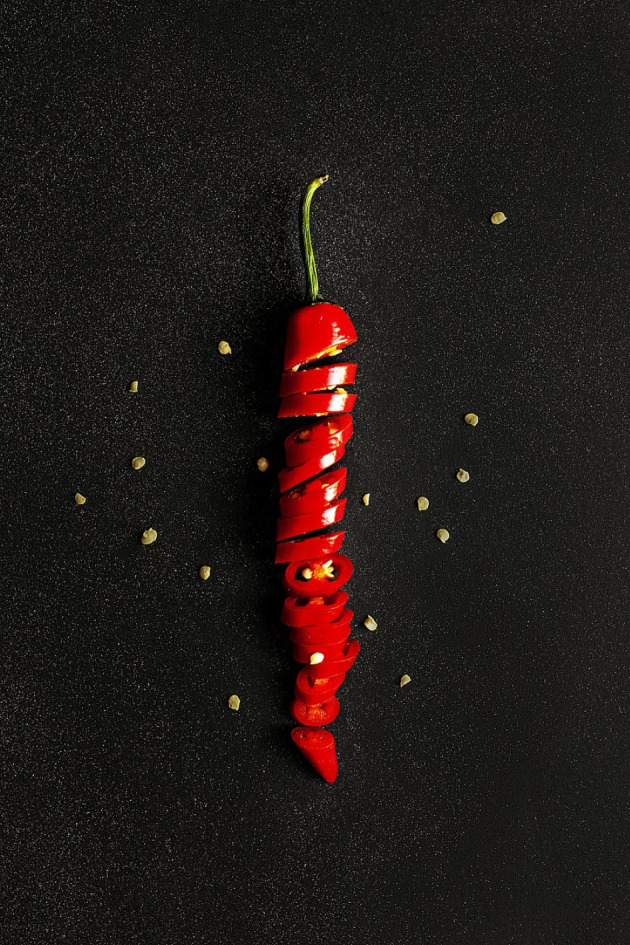
**Some naturally occurring compounds are perceived as having temperature. Capsaicin is responsible for the hot sensation associated with eating chillies.** Image reproduced under the terms of the Pixabay License.

Julius and, independently, Patapoutian began to investigate the sensory properties of additional members of the TRP family in mammals. They found that TRPM8 is both cold responsive and responsible for the cooling sensation of menthol and icilin (McKemy et al., 2002; Peier et al., 2002), whereas TRPA1 is cold responsive and senses horseradish, garlic, wasabi and mustard (Story et al., 2003). TRP channels have since been found to underpin a diversity of sensory functions in nature. Some snakes use a TRPA1 orthologue as an infrared detector, whereas camels and some ground squirrels have evolved a variant TRPV1 enabling them to withstand significantly elevated environmental temperatures (Laursen et al., 2016).

A multitude of translational opportunities have arisen from these discoveries. Because of the highly addictive nature of narcotic painkillers, pharmaceutical companies have identified TRP channels as attractive drug targets for pain management. These translational efforts were facilitated by the structural resolution of TRPV1 in 2013 (Koivisto et al., 2021). Just as moderate heat can help soothe muscle or joint pain, whereas noxious heat can inflict pain, both agonists and antagonists of TRP channels could have therapeutic potential. For example, a non-pungent TRPV1 agonist, MRD-652, has recently shown promise in a mouse model; resiniferatoxin, another capsaicin analogue, has proven efficacious in relieving pain in dogs with bone cancer. Clinical trials of first-generation TRPV1 antagonists in humans were largely unsuccessful. Some were even terminated early due to dangerous side effects, including burn injuries because of impaired noxious heat perception. However, a second-generation drug, mavatrep, has recently demonstrated efficacy against osteoarthritic pain with more tolerable side effects (Koivisto et al., 2021).

A decade after Julius first described TRPV1, Patapoutian and his co-workers at the Scripps Research Institute (La Jolla, CA, USA) embarked on a similar journey to identify the mechanical sensors that enable neurons to respond to physical touch. They first identified a mouse neuroblastoma cell line that was sensitive to mechanical force, and, hypothesizing that a cation channel is probably responsible, they made a shortlist of 72 known channels or proteins of unknown function that were enriched in the cell line. In a technical tour de force that I was fortunate to witness firsthand, Patapoutian's team systematically knocked down expression of each channel in the cell line using small interfering RNA (siRNA), and then captured electrophysiological responses to pressure stimulation. Over a year of constant experimentation passed; time after time, they observed no effect. Then, after knocking down their final candidate protein of unknown function, they recorded a dramatic reduction of inward current in response to pressure. They next expressed the channel in embryonic kidney cells, demonstrating that it confers sensitivity to mechanical force. Having proven both necessity and sufficiency, in 2010, Patapoutian's team reported the identification of the first mammalian mechanosensing ion channel, which they named PIEZO1 after the Greek term for pressure (Coste et al., 2010).

Patapoutian quickly realized that vertebrate species have a second, homologous touch-sensitive channel, PIEZO2, and focused his lab's efforts on understanding their respective roles in mediating the rich complexity of somatosensation *in vivo*. *Piezo1* knockout mouse embryos die at mid-gestation due to defects in vascular remodelling. This process relies on the appropriate detection of shear stress from blood flow, via Piezo1 acting in endothelial cells (Ranade et al., 2014a). Tissue-specific *Piezo1* knockout and gain-of-function mouse lines have uncovered additional mechanosensory roles in would healing, macrophage phagocytic activity, and erythrocyte turnover and stability.

In contrast, *Piezo2* knockout mice are viable. Phenotypic analysis revealed a severe deficit in light-touch sensation and a more minor impact on response to noxious mechanical stimuli (Ranade et al., 2014b). These mice also display impaired bladder control, whereas humans with loss-of-function mutations in PIEZO2 lack bladder-filling sensation and report atypical urinary voiding habits (Marshall et al., 2020). Mice lacking *Piezo2* in prociceptive neurons are uncoordinated with unusual limb movements; in the outer hair cells of the cochlea, *Piezo2* is necessary for ultrasonic, but not low-frequency hearing in mice (Li et al., 2021). *Piezo2* in vagal sensory neurons is critical for sensing airway stretch and lung inflation (Nonomura et al., 2017), demonstrating its role in mediating internal and external pressure-sensitive processes.

The interplay between the PIEZO channels was revealed in a stunning 2018 study into the molecular basis of baroreflex: the mechanism that maintains blood pressure at near-constant levels. Patapoutian and colleagues conditionally ablated both *Piezo1* and *Piezo2* in specific sensory ganglia, which resulted in the abolition of baroreflex, resulting in increased blood pressure variability (Zeng et al., 2018). More recently, Patapoutian and others have extended studies of PIEZO function into other model species, including *D. melanogaster*, *Caenorhabditis elegans* and *Arabidopsis thaliana*. These bodies of research undoubtedly place PIEZO channels at the very centre of eukaryotic somatosensory systems, critical for the perception of pain, touch, shear stress, body position and a broad range of internal homeostatic processes.

The genesis of some Nobel prizes can be traced to single moments of serendipity, inspiration or even luck (most famously, the 1993 Nobel winner Kary Mullis described his idea for PCR as an “accident”). The discoveries of TRP and PIEZO channels stand out as examples of another type of Nobel: exceptional curiosity-driven research borne out of persistence and tenacity, coupled with technical excellence. From cellular screens through mouse models to human genetics and translational medicine, for those interested in mechanisms of disease these awards deserve celebration.

**Some like it hot**

The recent COVID-19 pandemic has brought to the fore a further application of Julius and Patapoutian's foundational research into temperature sensation. Individuals who have lost their sense of smell and taste, disorders known as anosmia and ageusia, respectively, frequently report a severe disinterest in food due to their inability to fully perceive its flavour. A similar phenomenon is associated with aging, whereby diminished chemosensation can result in undernourishment and cachexia. These conditions are not uncommon, but are relatively underappreciated, even among medical professionals. This changed in 2020, almost overnight, when loss of smell and taste was recognized as the most common symptom of so-called ‘long COVID’. Activation of TRP channels through careful modulation of food with spice and temperature appears to be an effective strategy to combat the poor appetence associated with aging and chemosensory disorders in both humans and animals (Aschenbrenner et al., 2008; Eyre et al., 2021).
